# Patient education and follow-up as an intervention for hypertensive patients discharged from an emergency department: a randomized control trial study protocol

**DOI:** 10.1186/s12873-015-0052-3

**Published:** 2015-12-21

**Authors:** Julie Gleason-Comstock, Alicia Streater, Joel Ager, Allen Goodman, Aaron Brody, Laura Kivell, Aniruddha Paranjpe, Jasmine Vickers, LynnMarie Mango, Rachelle Dawood, Phillip Levy

**Affiliations:** Department of Family Medicine & Public Health Sciences, School of Medicine, Wayne State University, 3939 Woodward Ave., 48201 Detroit, MI USA; Cardiovascular Research Institute, School of Medicine, Wayne State University, 421 E. Canfield St., 48201 Detroit, MI USA; Center for Urban Studies, Wayne State University, 5700 Cass Ave., 48202 Detroit, MI USA; Department of Economics, Wayne State University, 656 W. Kirby St., 2074 FAB, 48202 Detroit, MI USA; Department of Emergency Medicine, Wayne State University, 4201 St. Antoine, UHC-6G, 48201 Detroit, MI USA

**Keywords:** Education of patients, Hypertension, Emergency medicine, Family practice

## Abstract

**Background:**

Persistently elevated blood pressure (BP) is a leading risk factor for cardiovascular disease development, making effective hypertension management an issue of considerable public health importance. Hypertension is particularly prominent among African Americans, who have higher disease prevalence and consistently lower BP control than Whites and Hispanics. Emergency departments (ED) have limited resources for chronic disease management, especially for under-served patients dependent upon the ED for primary care, and are not equipped to conduct follow-up. Kiosk-based patient education has been found to be effective in primary care settings, but little research has been done on the effectiveness of interactive patient education modules as ED enhanced discharge for an under-served urban minority population.

**Methods/Design:**

Achieving Blood Pressure Control Through Enhanced Discharge (AchieveBP) is a behavioral RCT patient education intervention for patients with a history of hypertension who have uncontrolled BP at ED discharge. The project will recruit up to 200 eligible participants at the ED, primarily African-American, who will be asked to return to a nearby clinical research center for seven, thirty and ninety day visits, with a 180 day follow-up. Consenting participants will be randomized to either an attention-control or kiosk-based interactive patient education intervention. To control for potential medication effects, all participants will be prescribed similar, evidenced-based anti-hypertensive regimens and have their prescription filled onsite at the ED and during visits to the clinic. The primary target endpoint will be success in achieving BP control assessed at 180 days follow-up post-ED discharge. The secondary aim will be to assess the relationship between patient activation and self-care management.

**Discussion:**

The AchieveBP trial will determine whether using interactive patient education delivered through health information technology as ED enhanced discharge with subsequent education sessions at a clinic is an effective strategy for achieving short-term patient management of BP. The project is innovative in that it uses the ED as an initial point of service for kiosk-based health education designed to increase BP self-management. It is anticipated findings from this translational research could also be used as a resource for patient education and follow-up with hypertensive patients in primary care settings.

**Trial registration:**

ClinicalTrials.gov Registration Number: NCT02069015. Registered February 19, 2014.

## Background

As a leading risk factor for cardiovascular disease, high blood pressure (BP) costs an estimated $47.5 billion annually in health-care expenditure [1]. Prevalence of hypertension among U.S. adults in 2003–2010 was 30.4 % (66.9 million) and an estimated 53.5 % (35.8 million) of these did not have their blood pressure under control. About 90 % of those with uncontrolled hypertension had a usual source of health care, insurance, and had visited a physician in the past year, suggesting missed opportunities for improving BP control [2]. Additionally, African-American adults, the clinic population for this study, have the highest prevalence of hypertension (44 %) in the United States as well as higher disease prevalence and consistently lower blood pressure control than Whites and Hispanics [3].

While physicians prescribe antihypertensive medications, a critical component of effective BP control rests with the individual. In one study, awareness, knowledge, and attitudes were more important than medication costs in achieving BP control [4]. Other individual factors such as chronic stress may be related to hypertension prevalence and BP control, especially for African-Americans [5]. Along with individual factors, environmental factors play a major role in BP control. For example, neighborhood disadvantage, measured by an index that includes the number of households living below the poverty line, has been highlighted as a factor that is significantly associated with poor blood pressure control in African-American older adults [6].

Because of the increasing prevalence of hypertension across ages, particularly among African-Americans, strategies to address blood pressure control are a critical public health issue [7, 8]. Population-based initiatives for hypertension prevention and control that leverage multiple community resources and address individual and socioeconomic factors are well positioned to have far reaching impacts on public health and are a high priority [9, 10]. Within the context of public health and the epidemiological paradigm of patient (host), emergency department (agent) and health service delivery (environment), the Haddon matrix has been further adapted to address the core elements of utilizing an urban emergency department as a portal for research and practice of population-based hypertension screening, intervention and delivery [8] (Table 1).

**Table 1:** Population-based systems approach to hypertension control

	Patient (Host)	Emergency department (Agent)	Health service delivery (Environment)
Precedent	Health belief/behavior	Treatment protocols	Public transportation
	Socioeconomic status	Physician knowledge	Economic climate
	Health Literacy	Case management	Educational opportunities
	Perceived disease severity		
Intervention	Treatment preferences	Patient management	Proximity to primary care
	Health status	Practice patterns	Quality of health services
	Healthcare coverage	Competing priorities	Contextual empathy
	Perceived level of risk	Patient discharge	Patient education
Antecedent	Self-efficacy	Referral to chronic care	Health education
	Clinical outcome	Provision of follow-up	Health information technology
			Access to medical care

Adaptation of the Haddan Matrix from Levy and Cline, 2009 [11]

Highly activated patients take more responsibility and acquire disease-specific knowledge and skills, thus promoting self-management and facilitating effective interactions with the health care system while also encouraging engagement in healthy behaviors [11–13]. Studies suggest that increased patient activation is strongly associated with a variety of self-management behaviors; however, there is little understanding of what interventions will improve activation [14]. Motivation and a sense of priority for patients to perform challenging self-care activities and implement discharge instructions may also play significant roles [15].

These factors are addressed in the Information-Motivation-Behavior (IMB) model, an evidence-based comprehensive health behavior change framework that has been applied to understanding engagement in a variety of health behaviors [16–19]. Patient education information includes knowledge about risk factors and behavior to modify risk. Motivation encompasses personal attitudes and beliefs, social norms and support systems. Behavioral skills include learning the specific skills needed to facilitate lifestyle modification. Self-efficacy provides the foundation for enacting these skills; patients are more likely to succeed if they self-monitor/evaluate their lifestyle progress, such as in weighing themselves regularly [20]. Barriers play an important role, influencing all three elements, but particularly personal motivation [17, 18, 21]. To be successful, interventions should focus on *modifiable* behaviors which should result in positive behavioral change [22].

Within the context of this modified IMB model, patient education delivered via information technology, i.e., interactive kiosks, can be a useful tool to promote hypertension control. Touch screen kiosks have been used with success as a provider-driven approach to deliver health education, improve knowledge, promote self-assessment, and monitor BP [23–30]. Importantly, those at greatest risk for poor BP control (i.e., underserved and minority populations) have reported satisfaction with interactive kiosk use [26, 27, 31], making it a viable option for studies targeting hypertension control in such groups.

**Fig. 1:**
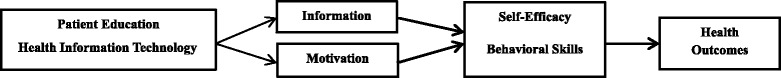
Kiosk-based patient education as enhanced discharge: IMB conceptual model

With the IMB model as our conceptual framework (Fig. 1), the proposed study will examine the application of patient education/health education delivered through health information technology to improving BP control and self-monitoring behavior. In the form of a touch screen interactive kiosk, participants in our study will receive a self-paced series of brief informational sessions about hypertension and guided through the process of taking their own BP using the attached blood pressure monitor. These multiple brief sessions are expected to increase motivation and behavioral skills. Using the kiosk to self-monitor and track their progress repeatedly is also expected to increase self-efficacy for using behavioral skills leading to increased patient activation and medication adherence for the health outcome of blood pressure control.

Results from a prior investigation by our research team in a clinic population of underserved, largely African-American adults indicated that brief health education sessions delivered by a touch screen kiosk with an attached BP monitor resulted in an overall change in BP that was statistically significant. In this feasibility study, at three month follow-up, 8 of the 25 participants (32 %) with uncontrolled BP at baseline had their BP under control (i.e., at or below 130/80) [20, 32]. These results paralleled other health studies, including one focused on diabetes education, in which participants reported that the health information technology delivered was equal or better than that received from healthcare providers because the information contained both audio and text, was step-by-step and logical, under their control, and they were able to learn something new in a limited time [33, 34].

Approximately 25 % of ED patients nationally have hypertension and 46 % of them are unaware of being hypertensive [35, 36]. The present study focuses on ED patients with uncontrolled hypertension the point of discharge. A previous study done by our team with African-American patients within this population indicated that subclinical hypertensive heart disease was highly prevalent (90.6 %), suggesting the need for focused efforts to reduce pressure-mediated consequences of hypertension [7]. Thus, the ED is an appropriate entry point to identify high-risk patients for whom our proposed intervention would be most beneficial.

## Methods

### Study design and aims

Achieving Blood Pressure Control Through Enhanced Discharge (AchieveBP) is a behavioral patient education intervention. It is a longitudinal, randomized controlled study in which ED patients with uncontrolled hypertension receive either an educationally enhanced or a standard discharge procedure. Study outcomes are assessed at baseline, 30, 90, and 180 days post ED discharge.

The design and conduct of the study adheres to the Consolidated Standards of Reporting (CONSORT) guidelines [37]. The study was approved by the Wayne State University Medical Institutional Review Board (M1, IRB#050213M1F) on 23 July 2013, and is registered with ClinicalTrials.gov (#NCT02069015). The study is administered through the Department of Family Medicine & Public Health Sciences in partnership with the Department of Emergency Medicine at the Wayne State University School of Medicine. Results of this study will be disseminated via scientific forums, including peer-reviewed publication and presentations at national and international conferences.

The primary aim of the study is to determine if integrating kiosk-based patient education via health information technology into the ED discharge process, coupled with additional educational sessions outside the ED will improve BP control compared to the usual discharge process. The secondary aim is to explore the relationship between patient activation, self-management behaviors (e.g., BP medication adherence), and health care utilization (i.e., return hospital emergency department visits). Cost effectiveness of the intervention will be assessed as well.

### Study population

Adults (age > 18 years) capable of utilizing the kiosk who have self-reported histories of hypertension and present with uncontrolled BP to the ED (>140/90 for non-diabetics and >130/80 for diabetics) will be targeted for recruitment. ED staff, familiar with the study eligibility criteria, will provide initial screening to identify potential study participants. As primary exclusion criteria, those patients with a diagnosis of end-stage renal disease, cognitive impairment, or requiring hospitalization after ED discharge will not be eligible for study participation.

### Consent and Randomization

**Fig. 2:**
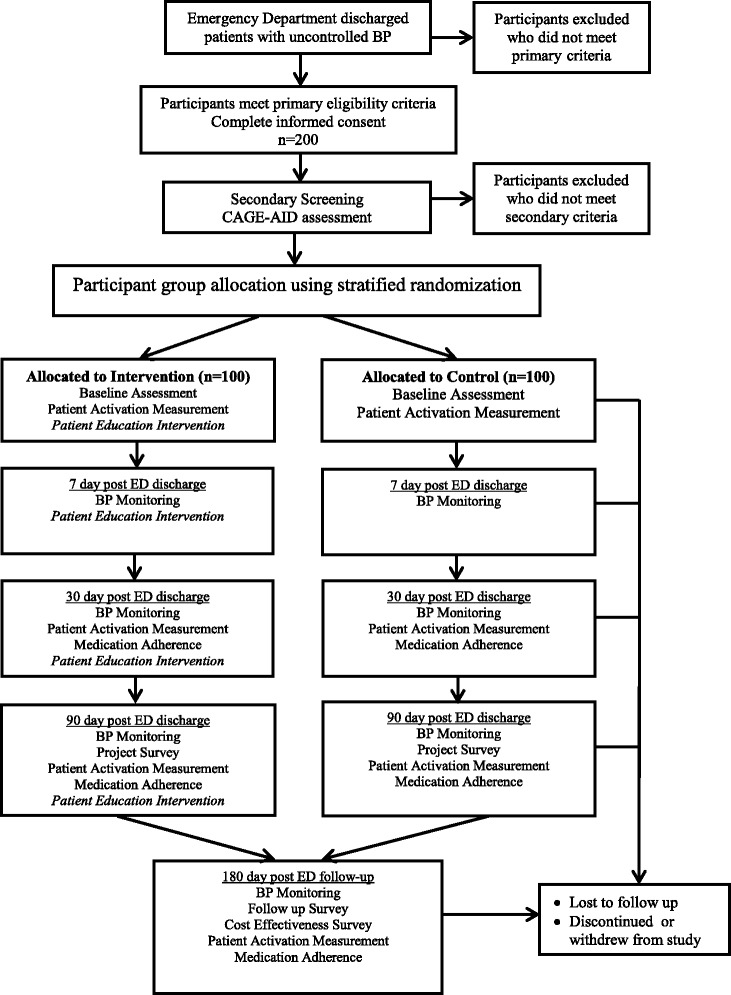
Study flow chart

Figure 2 shows the flow of the participants through the study. Pre-screening will be conducted by trained research assistants. Prior to consent, patients will be informed the enrollment process could take about an hour to complete. Once identified, research staff will meet with the potential participants to describe the study, answer questions, and obtain consent. After signed consent, project staff will use the CAGE questionnaire to screen for substance abuse disorders. Those who score two or more will be considered ineligible and dropped from the study. Up to 200 adults will be enrolled (i.e., 100 per study arm with oversampling by 50 % to account for attrition). A second BP reading will be obtained prior to randomization to verify that the high BP readings taken during initial screening remain at the eligibility level.

Given the differential definition for blood pressure control, a stratified random sampling procedure will be used, based on self-reported diabetic diagnosis. A block randomization schedule will be developed using a separate computer-generated random number table for diabetic and non-diabetics. Once diabetic status is determined, research staff will use the appropriate table to assign the participant to one of two study arms:

• Standard discharge (Attention control group) who will receive the ED’s usual discharge instructions for follow-up care. These materials typically include an accounting of major procedures and tests performed during the emergency department visit, principal diagnosis at discharge/chief complaint, patient instruction, follow-up care and medication/prescription.

• Intervention group, who, in addition to receiving the standard printed materials, will also receive a hypertension curriculum delivered via a touch screen kiosk with attached blood pressure monitor.

### Study procedures

Once randomized, participants will complete their baseline assessments at the ED. Participants randomized into the intervention arm will be shown how to use the kiosk to answer questions and will complete the first health education module before leaving the ED.

**Fig. 3:**
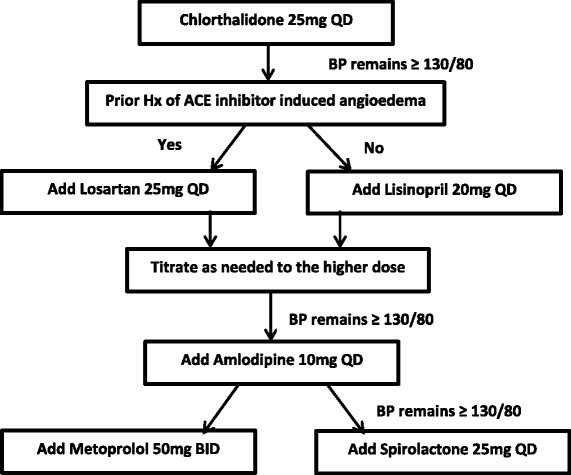
Anti- hypertensive regimen. Note: Medications are prescribed in the absence of any contraindications

To control for medication effects on blood pressure readings, all participants, regardless of study arm, will be prescribed a similar, evidence-based antihypertensive regimen [38], according to the algorithm illustrated in Fig. 3. Additionally all participants will receive an initial seven day medication supply at the conclusion of their ED visit. Participants on existing antihypertensive medication will have their medication adjusted to be in keeping with the study protocol. BP medication titration and side effect monitoring will occur at 7, 30, and 90 day post enrollment. All participants will receive primary care referrals as appropriate.

The study will be conducted by a multi-disciplinary team of trained emergency clinical medicine and public health researchers in two urban health environments: an emergency department and an academic clinical research center. Under the supervision of a research physician, patient screening, enrollment, baseline assessment and the first education intervention will be conducted by research assistants at the emergency department. The research physician will then review medication orders and provides instructions for the participant at enrollment. All subsequent study visits will occur at the clinical research center (CRC) about one mile away. At the beginning of each visit, the research physician and his clinical research team will review medication side effects, conduct pill counts and provide medication refills at seven, thirty and ninety day visits, with a three month prescription at the 180 day follow-up. Graduate level public health students will conduct post and follow-up interviews and facilitate patient education for the intervention group.

To promote retention, reminder calls and/or email will be done several days in advance and contact information will be updated at each visit. Both groups will be encouraged to monitor their blood pressure on their own and follow-up with a primary care physician. Participants missing visits and not responding to up to three reminder communication will be considered lost to follow-up. Participants will be withdrawn from the study for reasons such as withdrawing consent or moving too far away.

### Intervention

An evidence-based patient education hypertension curriculum, developed by American TeleCare, Inc., will be provided through a portable touch screen kiosk (Aviva® 200: InLife™ XP Patient Monitor/LifeView™ Video Patient Station). The series of four modules are designed to provide patient education about BP control and to help build lifestyle decision-making skills. The hypertension curriculum, which was used in the investigator feasibility study, was developed in 2006 and revised in 2011. For the present study, the first kiosk session will be completed in the ED. A second kiosk located in the research facility will be used for three subsequent sessions to be completed at the 7, 30, and 90 day visits.

Research staff will show participants how to use the kiosk and will remain close by to answer questions that may arise. If desired, the kiosk can be set to read the material aloud and participants can control the pacing of the material by touching the screen when they are ready for the next item. Once the session is completed the kiosk will send the encrypted information to a secure place on the ATI server for storage and review by the research team.

The beginning of each session walks the participant through the steps of taking his/her own BP using the attached monitor. The kiosk automatically records the reading and asks a series of questions about factors that could influence the readings (e.g., resting period, time since last consumed beverage and type of beverage). The measurement is followed by a series of hypertension health facts and associated true/false questions. If a participant answers a quiz item incorrectly, the associated health fact is repeated to reveal the correct answer. Flesch-Kincaid reading level [39] for the sessions is grade level 5.9. The first session, which will be provided at the baseline emergency department discharge visit is “What is Hypertension?” containing eight health facts and two quiz items. Subsequent topics to be covered during the follow-up visits include hypertension risk factors, suggestions on how to modify risks under their control, and the complications of uncontrolled BP. Although the number of health facts and quiz items vary by module, the average session will take about fifteen minutes. The health education modules will include definitions of blood pressure and hypertension, hypertension risk factors, the effects of drug and alcohol use on blood pressure, and the complications of poor blood pressure control. Sample health fact and quiz items are shown below in Table 2.

**Table 2:** *Achieve*BP patient education kiosk modules

Session	Module/Topic	Content example
1. Baseline	Blood Pressure and Hypertension Definitions	"Hypertension" is the medical term for high blood pressure. A person with high blood pressure is sometimes described as "hypertensive."
	Essential vs. Secondary Hypertension	Normal blood pressure is a systolic pressure of 120 or less. "Systolic" is the top number in a blood pressure reading.
	Systolic and Diastolic Normal Readings	
2. Seven Day	Hypertension Risk Factors	High blood pressure has many risk factors. Some you can't control. Family history - High blood pressure tends to run in families.
		Other risk factors for high blood pressure are within your control: Too much sodium (salt) in your diet which can cause your body to retain fluid, which increases blood pressure.
3. Thirty Day	Tobacco and Alcohol Use	If you drink more than moderate amounts of it, alcohol can actually raise blood pressure by several points. It can also reduce the effectiveness of high blood pressure medications.
	Caffeine Use	
		Cut back on caffeine. The role caffeine plays in blood pressure is still debatable. Drinking caffeinated beverages can temporarily cause a spike in your blood pressure, but it's unclear whether the effect is temporary or long lasting.
4. Ninety Day	BP Complications	Hypertension is frequently called the "silent killer" because it rarely causes symptoms. This is dangerous because untreated hypertension can lead to strokes, heart attacks, kidney disease, and vision loss.
	Complications of Uncontrolled Blood Pressure	
		Very high blood pressure is dangerous. You should call your Healthcare Provider anytime day or night, if your resting systolic blood pressure is over 180 or your resting diastolic blood pressure is over 110.

### Study outcomes

#### Primary outcome measure

The primary health outcome measure is short-term patient BP control. Blood pressure will be assessed using the BpTRU device (Smiths Medical PM, Inc; Waukesha, WI) which provides up to six automated measurements with initiation of the process by the patient rather than a health care provider. Blood pressure will be taken using this device at all assessment periods. Both the difference in the actual BP readings and the proportion of persons in each group achieving BP control at 180 days will be used to assess study outcomes. BP control is defined as at or below 140/90 for non-diabetics and at or below 130/80 for diabetics.

#### Secondary outcome measures

Secondary outcome measures include patient activation, medication adherence, health-related quality of life, and cost effectiveness. In regard to cost-effectiveness, to understand the financial burden associated with study participation, we will conduct a brief survey with participants at the completion of the study about costs they have incurred. These data will be combined with additional measures (e.g., ED and hospital recidivism) compiled by review of hospital records to derive estimates of the cost effectiveness of a kiosk-based approach to ED discharge, from a public health perspective.

Patient activation will be measured by The Patient Activation Measure (PAM) [40], a unidimensional thirteen item scale assessing patient knowledge, skill and confidence in self-management behaviors. Patients rate how strongly they agree/disagree with each item. Besides obtaining an activation score, responses can be used to stage individuals along an activation continuum: (1) believes active role is important, (2) confidence and knowledge to take action, (3) taking action, and (4) staying the course under stress. All participants will use the kiosk to complete the tool at baseline, 90, and 180 days post enrollment.

Medication adherence using the Morisky Medication Adherence Scale (MMAS) will be used to assess self-management behavior. The eight item MMAS, a self-report assessment tool shown to have a statistical association with antihypertensive medication refill rates, will be completed at 30, 90 and 180 days [41], also through the kiosk. Clinical research staff will conduct pill counts as part of medication monitoring at each follow-up appointment. Using fixed denominators based on a known quantity of medication dispensed at the prior evaluation, drug-specific and overall measures of antihypertensive therapeutic intensity will be derived.

A cost-effectiveness approach will be used to compare health outcomes (BP control) between the control and intervention groups [42]. The analysis will assume the objective of BP control is desirable even if the benefits have not yet been evaluated in monetary terms. The cost-effectiveness approach can be a useful step towards undertaking a cost-benefit study [43]. Because the study ultimately seeks to determine if an enhanced discharge can reduce ED return visits, this analysis will focus on the cost-saving comparison between groups as it relates to ED usage solely as related to hypertension. Clinical staff and hospital costs incurred at the ED visit as well as costs associated with follow-up (facility and staffing costs) will be measured. Participants will complete a brief survey about time and money costs they incurred from participating in the intervention study [44]. Additional information such as the number and reasons for return visits to the ED will be obtained from medical records.

### Statistical analysis

The OnCore Clinical Trial Data Management System, which is internet accessible and HIPAA compliant, will be used for protocol management & data entry. Primary analyses will be conducted using IBM SPSS (v. 20). Initial analyses will consist of descriptive statistics including univariate and bivariate frequency distributions. Differences in baseline socio-demographic and medical variables between the two groups will be used as covariates if appropriate statistical assumptions are met. Tests of the hypotheses will use the intent to treat analysis and all available outcome data.

To assess if the intervention improves blood pressure control over standard discharge practices, differences between groups will be assessed using blood pressure as a continuous variable (i.e., changes in systolic/diastolic readings) and as a dichotomous variable (i.e., number of patients with controlled blood pressure.

A generalized linear mixed model analysis will be used to test differences between groups over time, using continuous blood pressure measures. Both groups are expected to show decreases in blood pressure over time but with greater decreases for the intervention group. Thus, for the continuous blood pressure measures, the major effect of interest will be the linear group x time interaction. We assume that decreases in blood pressure over the time periods will be roughly linear, although a quadratic time effect may also occur. Based on a standard deviation of 12 mmHg for systolic blood pressure, a clinically relevant decrease of 6 mmHg systolic blood pressure (i.e., d = .5), and a one-sided alpha of .05, the study will have adequate power (.80) with 50 subjects in each group. This is a somewhat conservative estimate which does not take into account repeated measures, blocking, or possible covariate adjustments, all of which would tend to increase power.

Group differences in decreased percentages of those with uncontrolled blood pressure will be compared at each time point. Percentages will be compared using the arc sine transformation. For power assessment, we assume a medium effect size of h = .5 [45], where h is the difference between arc sine values for the population proportions of the two groups. Illustrative pairs of proportions for h = .5 are 60–35, 65–40, 69–45, and 79–50. For these effect sizes, *n* = 50, and a one-sided alpha of .05, power would be .80. General linear models will be used to test the full range of assessment periods.

In addition to assessing the primary outcome, the project will assess two secondary hypotheses related to patient activation. Analysis of covariance using the baseline activation score as covariate will be conducted to test for significant differences in patient activation scores between groups at the 180 day assessment. Pearson correlations and subsequent multiple regression analyses will test the association between patient activation scores and engaging in self-monitoring of blood pressure, medication adherence scores, demographics, and number of subsequent emergency department and primary care visits. Logistic regressions will explore differences in participant study completion and in obtaining BP control.

## Discussion

Persistently elevated BP is a leading risk factor for cardiovascular disease development, making effective hypertension management a critical public health issue. Emergency departments have limited resources for chronic disease management, especially for underserved patients dependent upon the ED for primary care. Kiosk-based patient education has been found to be effective in primary care settings, but little research has been done on the effectiveness of interactive patient education modules as part of the ED discharge process.

The AchieveBP trial will determine whether using kiosk-based patient education as ED enhanced discharge with follow-up at a clinical research center is an effective strategy for achieving short-term patient management of BP. The project is innovative in that it uses the ED as a point of service for kiosk-based health education designed to increase BP self-management. It is anticipated findings from this translational research could also be used as a resource for health education and follow-up with hypertensive patients in primary care settings.
